# Ancient genomes reveal the genetic structure and population interaction in the Central Plains of China during the Eastern Zhou Period

**DOI:** 10.3389/fmicb.2025.1602625

**Published:** 2025-05-27

**Authors:** Xiyan Wu, Pengxiang Liu, Fei Yan, RongPeng Jin, Canshuo Zhong, Lin Wang, Ke Chen, Fan Yang, Linyi Nie, Jinteng Liang, Yawei Zhou, Baoxu Ding

**Affiliations:** ^1^School of History and Culture, Henan University, Kaifeng, Henan, China; ^2^The Biological Archaeology Laboratory, Henan University, Kaifeng, Henan, China; ^3^Sanmenxia Yangshao Cultural Research Center, Sanmenxia, Henan, China; ^4^School of Archaeology and Cultural Heritage, Zhengzhou University, Zhengzhou, China; ^5^School of Life Sciences, Zhengzhou University, Zhengzhou, Henan, China

**Keywords:** ancient DNA, Zhou Dynasty, Central Plains, Guo State, Shangshihe cemetery

## Abstract

The Eastern Zhou period (770–256 BCE) was a transformative era in ancient China, marked by intensified social stratification, frequent warfare, and increased population movements. The Western Guo State, as an important vassal state during the Western Zhou period, migrated eastward with King Ping of Zhou and was eventually conquered by the Jin State. Due to limited genomic data, the genetic history of the Guo State population remains unclear. The Shangshihe cemetery, located in Yima City, Henan Province, is hypothesized to be associated with the Guo State and provides an opportunity to understand the genetic dynamics of this period. In our study, we successfully obtained 13 ancient genomes from the Shangshihe cemetery. Our findings suggest notable maternal and paternal genetic diversity within the Shangshihe population, although this observation is limited by the small sample size. Population genomic analysis shows that the Shangshihe individuals are predominantly of Yellow River Basin-related ancestry, with minor contributions from Southern East Asian-related and Eurasian Steppe-related sources. This genetic profile reflects extensive interactions between the Central Plains and surrounding populations during the Eastern Zhou period. Additionally, while distinct Y-chromosome haplogroups were observed among individuals with different burial orientations, autosomal analysis did not detect significant genetic differentiation, indicating overall genetic homogeneity within the population. These results not only elucidate the genetic characteristics of the Guo State population but also provide a new genetic perspective on the population dynamics and cultural exchanges in the Central Plains during the Eastern Zhou period.

## Introduction

The Central Plains of China, often referred to as the cradle of Chinese civilization, have played a pivotal role in the development of ancient societies and the interactions among diverse ethnic and cultural groups (Zhang et al., 2023). This region witnessed the rise and development of numerous significant archaeological cultures, including the Peiligang culture in the early Neolithic period, the Yangshao culture in the middle Neolithic period, the Longshan culture in the late Neolithic period, and the Erlitou, Erligang, and Yinxu cultures during the Bronze Age. It also served as the core area of ancient dynasties such as the Western Zhou and Eastern Zhou (Cheng, 2022). These cultures not only laid the foundation for the development of Chinese civilization but also fostered extensive interactions and exchanges among different populations.

Recent ancient genomic studies have provided profound insights into the demographic history of the Central Plains, revealing long-term genetic continuity among Yellow River Basin populations, alongside significant gene flow from Southern China and Southeast Asia since the late Neolithic period (Ning et al., 2020). Notably, certain late Neolithic groups (such as populations from the Yuzhuang and Yangshaocun sites) exhibit marked genetic heterogeneity and do not cluster with local Longshan-culture-related populations (YR_LN), possibly due to the absence of additional Southern East Asian-related ancestry compared to Yangshao-culture-related ancestry (YR_MN) (Li et al., 2024; Fang et al., 2025). Analysis of Bronze Age individuals from the Erlitou culture at the Wangchenggang site indicates that they are direct descendants of YR_LN-related ancestry. Further studies have revealed that the genetic influence of YR_LN-related ancestry from the Central Plains extends across a wide geographic range, impacting not only the Shandong Peninsula (Du et al., 2024) but also the Sichuan Basin (Tao et al., 2023), the West Liao River Basin (Ning et al., 2020), as well as the Hexi Corridor (Xiong et al., 2024), the Yunnan-Guizhou Plateau (Zhu et al., 2024), and northern coastal regions (Wang B. et al., 2024). These findings establish the Central Plains as a key hub for ancient population migration and interaction, with its genetic legacy profoundly shaping the broader genetic landscape of ancient China.

The Zhou Dynasty represents the longest-lasting dynasty in ancient Chinese history, conventionally divided into the Western Zhou (1046–771 BCE) and the Eastern Zhou (770–256 BCE) periods (Khayutina et al., 2010). During the Western Zhou period, the Zhou kings consolidated central authority and defended against threats from surrounding groups by enfeoffing territories and power to vassal states (Zhang, 1935). The Eastern Zhou period began with the eastward migration of King Ping of Zhou, marking a shift in the political center (Zhao, 1991). This era was characterized by intensified social stratification, frequent wars among vassal states, and increased population movements, which intensified interactions between the Central Plains and surrounding populations. As a significant vassal state established during the Western Zhou, the Western Guo State was initially located in present-day Baoji City, Shaanxi Province. Following the eastward migration of King Ping of Zhou, the Guo State was also relocated to Sanmenxia City in the Central Plains (Ren, 2001). In 655 BCE, the Guo State was conquered by the Jin State, with its remnants likely continuing to flee further eastward (Peng, 2006). These historical transformations were accompanied by large-scale population movements and cultural exchanges. However, genomic research on the genetic structure and interaction patterns of populations in the Central Plains during the Eastern Zhou period remains insufficient, and the genetic history of the Guo State population is still poorly understood.

Here, we analyzed ancient genomes from 13 individuals excavated at the Shangshihe cemetery, located in Yima City, Henan Province. Based on the unearthed artifacts and burial practices, this site is hypothesized to be the cemetery of the Guo State elite, their families, and retainers who fled eastward after being conquered by the Jin State (Yang and Yan, 2020). By comparing these samples with previously published ancient genomic data from the region, we aim to uncover the genetic structure of the Guo State population and elucidate their population dynamics and interactions with neighboring groups during the Eastern Zhou period.

## Materials and methods

### Archaeological context

**FIGURE 1:**
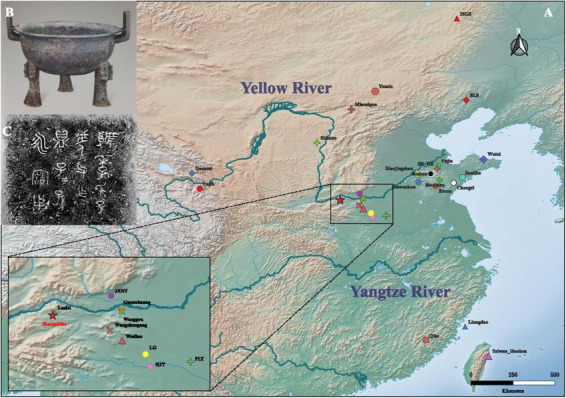
Geographic location of the Shangshihe cemetery. **(A)** Map showing the locations of published representative sites in China, including the Shangshihe cemetery marked with a red star. The region associated with the middle reaches of the Yellow River is highlighted and magnified for clarity. **(B)** A bronze vessel (ding) excavated from tomb M93 at the Shangshihe cemetery. **(C)** Inscriptions on the bronze vessel, provide evidence of a potential association between the Shangshihe cemetery and the Guo State. Adapted from Natural Earth, a public domain map dataset.

The Shangshihe Cemetery (110°50′47″E, 34°42′N), located in Shangshihe Village, Yima City, Henan Province, underwent two phases of excavation in 2017 and 2018 (Figure 1). The site yielded abundant cultural remains, including burials, horse pits, bronze vessels, pottery, jade artifacts, and stone tools (Yan et al., 2019). The tomb structures and burial goods assemblages are similar to those of the Guo State cemetery in Sanmenxia City. Based on the inscriptions on the bronze vessel and historical records, the cemetery is likely the burial site of the Guo State elite, their families, and retainers who fled eastward after the state was conquered (Yang and Yan, 2020). Previous studies have revealed clear social stratification at the site, with burial scale and orientation showing distinct patterns (Yang and Yan, 2020). Large and medium sized tombs are predominantly aligned north-south, consistent with the Guo State cemetery, while smaller tombs are mostly oriented east-west. These findings support the hypothesis that the Shangshihe Cemetery is linked to the Guo State during the Eastern Zhou period. We collected teeth and petrous bones from the human remains. A total of 13 individuals from the Shangshihe cemetery were selected for ancient DNA analysis.

### Ancient DNA extraction, library preparation, and sequencing

DNA extraction and library preparation were conducted following established protocols in a dedicated ancient DNA facility at Henan University, China (Korlević et al., 2015). Human remains were decontaminated using 75% ethanol, followed by a 5% NaClO wash, and then exposed to UV light for 30 min per side. Tooth samples were processed into powder using a dental drill (Strong 90). Petrous bones were ground into fine powder using an automated grinder (JXFSTPRP-24L) after the outer layers were removed with a sandblaster machine (Renfert, Germany). DNA was extracted from 50 to 120 mg of bone powder following Dabney’s protocol (Dabney et al., 2013). Double-stranded libraries were constructed using a published protocol with minor modifications, without uracil-DNA glycosylase (UDG) treatment (Meyer and Kircher, 2010). Briefly, DNA was blunt-ended using T4 PNK and T4 polymerase at 15°C for 15 min and 25°C for 15 min, followed by purification with the MinElute PCR Purification Kit (Qiagen) using TET buffer (Tween20, EDTA, Tris-HCl) for elution. Designed adapters were then ligated to the blunt ends, and a second purification was performed. The adapter fill-in step was performed using Bst DNA polymerase at 37°C for 20 min and 80°C for 20 min. Libraries were amplified with dual-indexing primers (P5 and P7) using Q5 DNA polymerase and then purified using AMPure XP Beads (Beckman Coulter). The final libraries were quantified using a Qubit 4.0 Fluorometer (Thermo Fisher Scientific) and sequenced on an Illumina NovaSeq 6000 platform at Novogene Co., Ltd., China.

### Sequence data processing and genotyping

The paired-end sequencing data were merged into single-end reads using AdapterRemoval v2.3.3 (Schubert et al., 2016). The merged reads were aligned to the human reference genome (hs37d5) using BWA v0.7.17 with the parameter “-n 0.01” (Li and Durbin, 2009). The aligned reads were converted to BAM format and sorted using Samtools v1.17 (Li et al., 2009). Duplicate sequences resulting from PCR amplification were removed using DeDup v0.12.8 (Peltzer et al., 2016). The BAM files were then indexed with Samtools. Coverage of the genome was assessed using Qualimap v2.2.2 (Okonechnikov et al., 2016), and ancient DNA damage patterns were evaluated with mapDamage v2.2.2 (Jónsson et al., 2013). Quality control was performed by filtering reads with a mapping quality of at least 30 using Samtools view (-q 30), followed by trimming of 10 bp from the ends with trimBam (Danecek et al., 2021). In addition, we evaluated mitochondrial contamination levels using Schmutzi (Renaud et al., 2015) and determined nuclear genome contamination rates in males with ANGSD v0.940 (Korneliussen et al., 2014). Genotype calling was performed using SAMtools mpileup with the parameters “-q30, -Q30” and pileupCaller,^1^ applying the “—randomHaploid” option to generate pseudodiploid genotypes through random sampling from the 1240K SNP dataset (Mallick et al., 2024). The resulting genotype data were then combined with worldwide genotype datasets, derived from the Human Origins (597,573 SNPs) panel^2^ and the 1240K (1,233,013 SNPs) panel.

### Genetic sex determination and uniparental haplotype assignment

Sex determination was performed by calculating the ratio of Y-chromosomal and X-chromosomal coverage relative to that of autosomal coverage (Fu et al., 2016). Mitochondrial haplogroups were determined by mapping trimmed and collapsed reads to the human mitochondrial reference genome (NC_012920.1). Consensus sequences were generated using the log2fasta tool from the Schmutzi package, followed by haplogroup assignment with HaploGrep v2.4.0 (Weissensteiner et al., 2016). For Y-chromosomal haplogroup identification, the Yleaf software was used (Ralf et al., 2018), specifically employing the “Yleaf.py” and “predict_haplogroup.py” scripts to generate results that include both ancestral and derived SNPs within the ISOGG Y-chromosome phylogenetic framework. To ensure accuracy, these key SNPs were examined using the Integrative Genomics Viewer software (Thorvaldsdóttir et al., 2013).

### Genomic analysis of homozygosity and kinship

Runs of Homozygosity (ROH) analysis serves as a genomic approach to identify continuous homozygous segments, providing insights into inbreeding and population bottleneck events. In this study, we employed hapROH^3^ to investigate the occurrence of consanguineous practices in the Shangshihe population (Huang and Ringbauer, 2022). For kinship estimation, we applied two different methods, including the Pairwise Mismatch Rate (PMR) and the Relationship Estimation from Ancient DNA (READ), to ensure robust relatedness inference (Kennett et al., 2017; Kuhn et al., 2018). The PMR method quantifies genetic divergence by calculating the ratio of mismatched SNP sites between paired individuals and normalizing this count by the total number of loci with available data for both. The READ method evaluates genome-wide similarity by measuring the proportion of discordant alleles across non-overlapping 1 Mb genomic windows, enabling precise estimation of kinship coefficients (Kuhn et al., 2018).

### Population structure analysis

To explore the genetic structure of the Shangshihe population, we conducted principal component analysis (PCA) on the Human Origins (HO) dataset using the smartpca v18140 tool from the EIGENSOFT package (Patterson et al., 2006), enabling the “lsqproject: YES” and “shrinkmode: YES” options. Ancient samples were projected onto principal components derived from these modern populations to reveal inter-population affinities. The PCA results were visualized using the ggplot2 package in R.^4^ Additionally, we performed an unsupervised ancestry analysis with ADMIXTURE v1.3.0 (Patterson et al., 2012) on the HO dataset. Prior to analysis, we excluded SNPs with a minor allele frequency below 1% (–maf 0.01) and applied linkage disequilibrium pruning in Plink v1.90 (Chang et al., 2015) with the parameters “–indep-pairwise 200 25 0.2.” The ADMIXTURE analysis was executed across K values from 2 to 20, and cross-validation (CV) errors were calculated to determine the optimal K value, with lower CV values reflecting enhanced model stability. The resulting ancestry proportions were subsequently visualized using the AncestryPainter tool (Feng et al., 2018).

### Allele sharing analysis

To investigate the shared genetic drift between the Shangshihe population and other groups, we calculated the outgroup f3-statistics and f4-statistics using the qp3pop v651 and qpDstat v980 programs from the ADMIXTOOLS package (Patterson et al., 2012). The outgroup f3-statistics were computed in the form f3(Mbuti.SDG; X, Shangshihe) with the “inbreed: YES” parameter enabled. Here, the Mbuti population from Central Africa served as the outgroup, while X represented various Eurasian populations. The top 30 populations with more than 10,000 SNPs, ranked by f3 values, were visualized using DataGraph v4.5.1. Additionally, the f4-statistics were performed with the parameter “f4mode: YES” on the model (Mbuti.SDG, Reference; X, Shangshihe). This model was used to explore additional gene flow between the Shangshihe population and either the reference groups or X populations. For this analysis, the reference groups comprised by ancient and modern Eurasian populations, while X consisted of ancient populations from diverse East Asian regions. We further employed the f4(Mbuti.SDG, YR_related; Shangshihe, ancient Eurasians) model to test whether ancient populations from the Yellow River Basin share more ancestry with the Shangshihe population. Standard errors were calculated using 5 cM block jackknifing, as implemented in the f3-statistics and f4-statistics analyses.

### Admixture modeling analysis

To determine whether different individuals from the Shangshihe population could be modeled as deriving from the same ancestral sources, we conducted pairwise qpWave tests using the qpWave v1520 program from ADMIXTOOLS (Patterson et al., 2012). A rank of 0 with a *p*-value greater than 0.01 suggests that the tested individuals share a consistent ancestry profile without evidence of distinct admixture events. Next, we performed qpAdm analysis to further explore the specific ancestry sources and their proportions, using qpAdm v1520 from the same package with the parameters “allsnp: YES” and “details: YES.” We set up a base outgroup, including Central African outgroup (Mbuti), indigenous Andamanese islanders (Onge), Western Eurasian hunter-gatherers (Loschbour), Ancient Northern Eurasian population (Botai), Neolithic farmers from Iran (Iran_N), Ancient Northeast Asian population (Shamanka), Early Asian lineage associated with the Jomon culture (Japan_Jomon), Early Neolithic population from Fujian (Fujian_EN), Indigenous Taiwan group (Ami), and early Neolithic population from Shandong (Shandong_EN). To evaluate competing admixture hypotheses, we implemented a rotation strategy, adding source populations to the base outgroup and iteratively running qpAdm to refine the ancestry proportions and assess model validity (Harney et al., 2021).

## Results

### Ancient genome-wide data from the Shangshihe cemetery

We extracted DNA from 13 human skeletal remains at the Shangshihe cemetery and performed shotgun sequencing. All individuals yielded sufficient endogenous DNA content (3.99–74.09%), resulting in autosomal coverage ranging from 0.02X to 0.93X and mitochondrial DNA coverage from 5.68X to 38.79X (Table 1; Supplementary Table S1). Analysis of the data revealed typical ancient DNA damage patterns, including C-to-T misincorporations at the 5′ end and G-to-A misincorporations at the 3′ end (Supplementary Figure S1), along with short average fragment lengths (ranging from 52.74 to 83.14 bp) (Table 1). Mitochondrial DNA contamination rates were below 5% for all individuals except SSH48, and X-chromosome contamination in male samples ranged from 0.65 to 3.24% (Supplementary Table S1). These characteristics confirm the authenticity and reliability of the genomic data obtained in this study. After excluding samples with high contamination (>5%) and low SNP counts (< 30,000) that overlapped with the 1240K SNP panel, the remaining individuals were used for downstream population genomic analyses.

**TABLE 1:** A summary of Shangshihe samples reported in this study.

Sample ID	Skeletal element	Number of reads sequenced	Endogenous DNA	Library length	Genetic sex	Coverage	MtDNA haplogroup	Y haplogroup	1240K SNPs
SSH14N	Petrous	60,133,296	41.20%	66.79	Male	0.4068	B4c1b2c	C2b1b	298,608
SSH19N	Petrous	59,184,184	32.39%	59.61	Male	0.2314	F2c	C2b1b	163,485
SSH21	Petrous	39,119,349	57.06%	83.14	Male	0.4876	A8	O2a	232,463
SSH38N	Petrous	51,631,926	41.01%	63.38	Male	0.3146	N9a1	N1a2	234,535
SSH43N	Petrous	84,640,298	45.47%	62.06	Female	0.6494	G2a’c	#	388,278
SSH48	Petrous	15,515,382	4.42%	52.74	Male	0.0095	N9a1	–	7,966
SSH60	Petrous	46,932,358	74.09%	65.6	Male	0.7469	D4a5	O2a	373,472
SSH65	Petrous	68,743,009	70.30%	36.42	Male	0.929	F1 + 16189	Q1a1a1a	367,231
SSH74	Tooth	57,532,391	15.36%	51.18	Male	0.1128	G2a1	N1b2	87,242
SSH82	Tooth	69,261,352	4.96%	61.35	Female	0.0549	D5a3	#	43,295
SSH94	Tooth	19,047,120	16.96%	67	Female	0.0595	B4a1c5*	#	42,848
SSH96	Petrous	26,289,906	3.99%	56.99	Female	0.0157	F2 + 16291	#	12,415
SSHM018	Tooth	11,002,488	25.32%	60	Female	0.047	C7	#	34,399

The “#” symbol in the Y haplogroup column indicates that the sample is female and therefore lacks Y haplogroup data.

The “*” symbol in the MtDNA haplogroup column for sample SSH94 (B4a1c5) denotes that this sample belongs to a basal lineage of the B4a1c5 haplogroup, meaning it does not belong to a more derived subgroup within B4a1c5. These annotations are accurate and consistent with the genetic data presented.

### Uniparental genetic analysis, kinship, and parental relatedness assessment

We successfully assigned the mitochondrial haplotypes for all individuals. A total of 12 mtDNA haplogroups were detected (Table 1), including A (A8), B (B4c1b2c, B4a1c5*), C (C7), D (D4a5, D5a3), F (F1 + 16189, F2c, F2 + 16291), G (G2a1, G2a’c), and N (N9a1). These haplogroups are common in East Asia and indicate high maternal genetic diversity within the Shangshihe population. Most of these haplogroups were found in various sites across the Yellow River Basin over different periods. For instance, haplogroup D4a5 was found in Middle Neolithic sites such as the Wanggou and Qingtai sites, as well as in Shandong_LN populations (Ning et al., 2020; Wu, 2024; Liu et al., 2025). Haplogroups N9a1 and G2a1 were identified in Late Neolithic sites including the Shimao and Taosi sites (Xue et al., 2022). Haplogroups B4c1b2c, D5a3, and F1 + 16189 were detected in the Lusixi site during the Iron Age (Ma et al., 2025). These findings demonstrate the long-term continuity of maternal genetic structure in the Yellow River Basin.

In addition, some haplogroups were also detected in ancient populations from Southern China. For instance, haplogroup B4c1b2a (a sister branch of B4c1b2c) was found in the Dayin and Xitoucun sites during the Late Neolithic period (Wang et al., 2020; Liu et al., 2021). Haplogroup C7 was identified in the Babanqincen and Gaohuahua sites from the historical period (Liu et al., 2022). These observations suggest significant interactions between the Shangshihe population and surrounding groups in the maternal lineage. Furthermore, seven male individuals were successfully identified with Y-chromosome haplogroups, all of which belonged to the East Eurasian types (C2b1b, O2a, N1a2, N1b2, and Q1a1a1a), exhibiting high paternal genetic diversity (Table 1). These haplogroups were also found in the contemporaneous Guanzhuang site (Wu et al., 2024). Collectively, these results suggest that the Shangshihe population has diverse paternal and maternal genetic origins.

We assessed the kinship relationships among Shangshihe individuals using two complementary methods (PMR and READ) to ensure the robustness of our results. Only samples with more than 30,000 SNPs were included in the kinship analysis, which revealed no close genetic relationships among the individuals (Supplementary Figure S2; Supplementary Table S2). We further evaluated parental relatedness by measuring the length of Runs of Homozygosity (ROH) for each individual. Our findings showed that five individuals (SSH19N, SSH38N, SSH43N, SSH65, and SSH74) exhibited ROH segments exceeding 4 centiMorgans (cM) (Supplementary Table S3). Among them, SSH65 and SSH43N also displayed a limited number of longer ROH segments (> 20 cM). However, with total ROH lengths remaining below 130 cM, there was no evidence of inbreeding or close intermarriage, such as between second cousins, within the selected samples (Supplementary Figure S3).

### The genetic structure of the Shangshihe population

**FIGURE 2:**
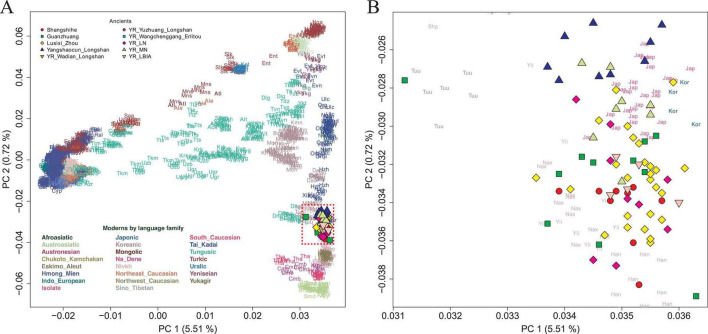
PCA analysis for Shangshihe individuals. **(A)** Ancient genomes from Shangshihe and other ancient individuals in the Yellow River Basin were projected onto the top PCs defined by present-day Eurasians. **(B)** A zoomed-in view focusing on the distribution of ancient individuals from the middle Yellow River Basin.

**FIGURE 3:**
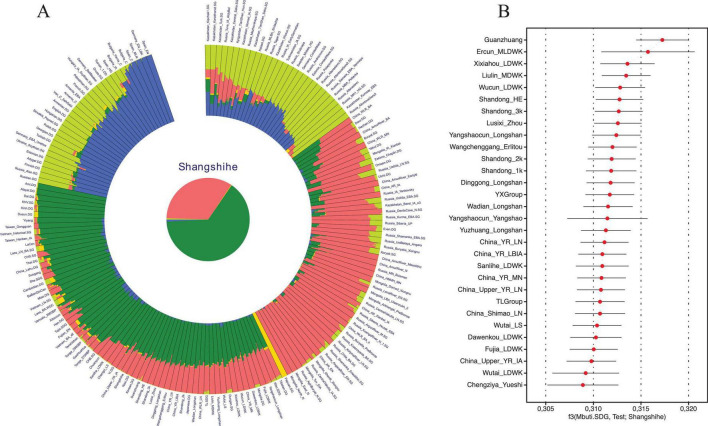
Genetic profile of the Shangshihe population. **(A)** Admixture analysis of the Shangshihe population and other populations from the “HO” dataset at K = 5. Each color represents a distinct ancestry component, illustrating that the Shangshihe population is primarily composed of Northern East Asian (pink) and Southern East Asian (green) ancestries. **(B)** Outgroup f3 statistics in the form of f3(Mbuti.SDG, Test; Shangshihe). The top 30 populations exhibiting the highest genetic drift with the Shangshihe populations are presented.

To characterize the genetic structure of the Shangshihe population, we employed principal component analysis (PCA) to visualize population structure through dimensionality reduction. Ancient samples were projected onto the genetic variation defined by present-day Eurasian individuals. We found that the Shangshihe population clustered with the modern Han Chinese population and ancient populations from the Yellow River Basin (Figure 2). Compared to Middle Neolithic populations in the Yellow River Basin (YR_MN), the Shangshihe individuals are shifted southward along PC2, clustering with Late Neolithic populations (such as YR_Wadian_Longshan, YR_LN), Bronze Age populations (such as YR_LBIA, YR_Wangchenggang_Erlitou), and Zhou dynasty populations (such as Lusixi_Zhou and Guanzhuang). To further refine our analysis, we conducted an additional PCA focusing on East Asian genomes. This revealed that Shangshihe individuals exhibit strong genetic similarities to ancient populations dating from the Bronze Age to Iron Age in the Central Plains of China (YR_LBIA) as well as modern Northern Han populations (Supplementary Figure S4). These findings were consistent with model-based unsupervised ADMIXTURE simulation analysis. Our analysis identified the model with the lowest cross-validation error at K = 5 (Supplementary Figure S5). Most populations in the Yellow River Basin were admixed with Northern East Asian-related ancestry and Southern East Asian-related ancestry (Figure 3A). The Shangshihe population and other Zhou dynasty populations (Lusixi_Zhou and Guanzhuang) displayed similar structure, with increased Southern East Asian ancestry compared to Neolithic populations. This indicates intensified genetic interaction between Central Plains populations and Southern East Asian populations after the Neolithic period.

Similar results were observed in the outgroup f3-statistics in the form of f3(Mbuti.SDG, X; Shangshihe). Larger f3 values indicate that the Shangshihe population shares more genetic drift with the test population. The strongest genetic sharing was observed with the Guanzhuang population, followed by various Yellow River Basin groups, including those from the Yangshao and Longshan cultures (Figure 3B; Supplementary Table S4). This points to a broad genetic continuity across Yellow River Basin populations over time. Additionally, the Shangshihe population shows a remarkably close relationship with historical-period and Dawenkou culture populations from Shandong, consistent with previous studies (Du et al., 2024). This suggests that Neolithic farmers from the middle Yellow River likely contributed genetically to populations downstream, highlighting regional gene flow within the basin.

### Intensified population interactions during the Eastern Zhou period

**FIGURE 4:**
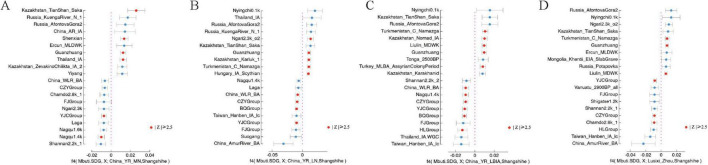
Symmetry F4 statistics to evaluate the genetic divergence between the Shangshihe population and Yellow River-related populations. **(A)** F4 Statistics test in the form of f4(Mbuti.SDG, X; China_YR_MN, Shangshihe). **(B)** F4 Statistics test in the form of f4(Mbuti.SDG, X; China_YR_LN, Shangshihe). **(C)** F4-Statistics test in the form of f4(Mbuti.SDG, X; China_YR_LBIA, Shangshihe). **(D)** F4-Statistics test in the form of f4(Mbuti.SDG, X; Lusixi_Zhou, Shangshihe). For each test, the 10 most positive and 10 most negative f4 values are presented. The statistically significant results (|Z| ≥ 2.5) are colored in red.

The genetic interactions influencing the Central Plains population during the Eastern Zhou period are reflected in the Shangshihe population. Our analyses using PCA, f3-statistics, and admixture modeling reveal a close genetic relationship between the Shangshihe population and YR-related populations. To further investigate potential gene flow between the Shangshihe population and other groups, we applied the f4 statistics in the form of f4(Mbuti, X; YR-related, Shangshihe). When YR-related populations were represented by the Guanzhuang population, and X included various Eurasian groups, the results of f4 statistics showed non-significant Z-values (|Z| < 2.5), confirming a close genetic relationship between Guanzhuang and Shangshihe populations (Supplementary Figure S6; Supplementary Table S5). However, when comparing the Shangshihe population to the Yangshaocun_Longshan population, we observed genetic influences from Southern East Asian populations, such as Tonga_2700BP, Vietnam_BA, and Laos_LN_BA populations, with Z-scores exceeding 2.5 (Supplementary Figure S6). Similarly, when the Shangshihe population was compared to the Longshan culture-related populations from the Yellow River Basin (e.g., YR_Wadian_Longshan), we observed distinct genetic differences (Figure 4; Supplementary Figure S6). Significant gene flow (Z-scores > 2.5) was detected with some Eurasian steppe populations (e.g., Kazakhstan_Nomad_IA.SG, Hungary_IA_Scythian, and Kazakhstan_TianShan_Saka.SG). Similar steppe-related genetic signatures were also observed when comparing Shangshihe with the Lusixi_Zhou population (Figure 4; Supplementary Table S5). These findings suggest that the Shangshihe population likely experienced gene flow with these diverse groups, demonstrating the genetic interactions during the Eastern Zhou period.

**FIGURE 5:**
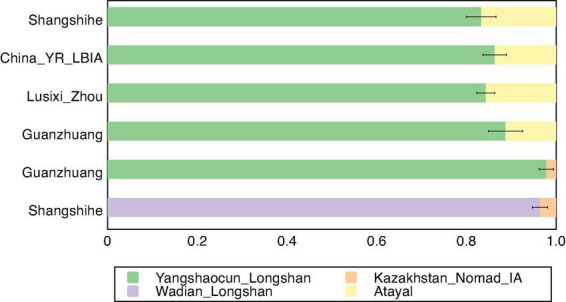
Ancestry proportions based on qpAdm analysis. The well-fitted 2-way qpAdm models for Shangshihe and previously published populations including China_YR_LBIA, Guanzhuang, and Lusixi_Zhou are presented. Detailed results are provided in Supplementary Table S6.

The population interactions were further reflected through qpAdm ancestry modeling. Shangshihe could be effectively modeled as a single-source population derived from YR-related groups in one-way models (Supplementary Table S6A). However, incorporating Southern East Asian (SEA)-related populations as an additional source, Shangshihe was modeled as approximately 89.3–91.8% YR-related ancestry (represented by YR_Yuzhuang_Longshan or Lusixi_Zhou) and 8.2–10.7% SEA-related ancestry (represented by Atayal or Thai) (Supplementary Table S6B). More specifically, we detected a subtle steppe influence, with Shangshihe comprising 96.4–98.8% YR-related ancestry (e.g., YR_Wadian_Longshan or Lusixi_Zhou) and 1.2–3.6% Eurasian steppe ancestry (e.g., Kazakhstan_Nomad_IA) (Figure 5). Notably, compared to the contemporary Lusixi_Zhou population, qpAdm results did not detect any Eurasian steppe ancestry (Supplementary Table S6C), highlighting the high genetic diversity of the Central Plains population during the Zhou Dynasty. These best-fitting admixture models align with the f4 findings, emphasizing diverse ancestral origins in the Shangshihe population.

In summary, the Shangshihe population exhibits a complex genetic profile characterized by dominant YR-related ancestry alongside influences from Southern East Asian-related and Eurasian steppe-related sources. This admixture pattern likely reflects intensified population interactions during the Eastern Zhou period, possibly driven by cultural exchange, population migration, or trade networks in the ancient Central Plains.

## Discussion

The Eastern Zhou period (770–256 BCE) was a transformative era in China, characterized by the gradual breakdown of the traditional feudal and clan-based systems established during the Western Zhou dynasty (Jin, 1956). During this era, population migration and frequent warfare significantly intensified population movements and fostered extensive interactions between the Central Plains and surrounding groups. The Shangshihe cemetery is one of the important cultural sites of the Eastern Zhou period. According to the tomb styles, inscriptions on bronze vessels, and artifacts, it suggests that the Shangshihe cemetery may be associated with the Guo State (Yang et al., 2021). This historical context provides a unique opportunity to understand population dynamics in the Eastern Zhou period.

In this study, we successfully obtained genome-wide data from the Shangshihe cemetery. Our findings show that the Shangshihe population exhibits a strong genetic affinity with populations from the Yellow River Basin. PCA and f3 statistics show close relationships with populations from the Late Neolithic period (e.g., YR_Wadian_Longshan and YR_LN), the Bronze Age (e.g., Wangchenggang_Erlitou), and the Zhou period (e.g., Guanzhuang and Lusixi_Zhou) in the Central Plains. The qpAdm model estimates that 89.3–91.8% of their ancestry comes from YR-related sources, reinforcing the idea of long-term genetic stability in this region. This finding is consistent with previous research that underscores the genetic continuity within the Yellow River Basin (Ma et al., 2025), which highlights its role as a stable core of Chinese civilization despite the significant social and political transformations during the Eastern Zhou period.

Our study also reveals increased population interactions during the Eastern Zhou period, as indicated by f4 statistics and qpAdm modeling. First, strong connections with the middle and late Dawenkou culture, as well as historical-period populations from Shandong (SD_HE), likely reflect migration and cultural spread within the Yellow River Basin, aligning with patterns of gene flow from the middle to lower reaches since the Neolithic (Wang F. et al., 2024). Additionally, genetic contributions from Southern East Asian-related ancestry were detected, indicating intensified interactions between the Central Plains and Southern East Asian populations after the Neolithic period. These findings align with previous archeological and genetic findings that show bidirectional cultural interaction and population movement, which have profoundly enriched the cultural landscape of the Central Plains region (Song et al., 2018; Wu et al., 2024). At the same time, we identified a small but notable Eurasian steppe component in the Shangshihe population, which was absent in the contemporary Lusixi_Zhou group. This steppe genetic signature likely reflects contact and gene flow with northern nomadic groups. During the Eastern Zhou, conflicts and cultural exchanges between Central Plains states and northern nomads were common, and the eastward migration of the Guo State likely facilitated gene flow with surrounding populations (Peng, 2006). Although the steppe contribution is minor, its statistical significance suggests higher genetic diversity in the Shangshihe population, potentially linked to its geographical location at the confluence of Central Plains agricultural civilization and the northern steppe culture.

The Shangshihe cemetery also provides insights into the social hierarchy and burial practices of the Eastern Zhou period. Previous studies have shown that individuals buried with a north-south orientation were typically interred in larger tombs with richer grave goods and exhibited a protein-rich diet, suggesting higher social status (Zhou et al., 2021). In contrast, tombs oriented east-west were generally smaller with fewer grave goods. These observations imply that the burial orientation and tomb size were significant markers of social status during this period. Our genetic analysis further reveals distinct Y-chromosome haplogroups among individuals with different burial orientations. For instance, haplogroups N and Q were observed in individuals buried with a north-south orientation, while haplogroups O and C were more common in those with an east-west orientation. However, this pattern should be interpreted cautiously, as it may be influenced by the small sample size and potential sampling bias. Despite these differences in Y-chromosome haplogroups, our analysis of autosomal DNA did not reveal significant genetic differentiation between individuals with different burial orientations. Using f4 statistics in the form of f4(Mbuti, X; Shangshihe_WE, Shangshihe_NS), we found no significant Z values (Supplementary Table S7), and qpWave modeling (*P* > 0.05) confirmed that individuals from both orientations could be traced to the same ancestral sources (Supplementary Figure S7). These findings tentatively suggest that while paternal lineages might reflect differences associated with social status, the overall genetic makeup of the population appears to have been relatively homogeneous, though this observation is limited by the small sample size. This homogeneity may be a result of the extensive interactions and gene flow that characterized the Eastern Zhou period, as evidenced by the presence of genetic contributions from both Southern East Asian and the Eurasian steppe populations. The lack of significant autosomal differentiation further suggests that burial practices and social status may not have led to long-term genetic isolation within the population. However, the current study is limited by the small sample size and low coverage in some samples, which may restrict the detection of subtle genetic signals. Future studies with larger samples, higher sequencing depth, and integration of additional archeological and historical data will help reveal the social mechanisms behind these genetic exchanges, providing a fuller understanding of the complex history of Central Plains populations during the Eastern Zhou.

In summary, our study sheds new light on the population structure and genetic dynamics of Zhou Dynasty inhabitants in the Central Plains. Our findings indicate that the genetic profile of the Shangshihe population is predominantly characterized by Yellow River-related ancestry, with minor contributions from Southern East Asian and the Eurasian steppe populations, reflecting enhanced interactions among the Central Plains populations during the Eastern Zhou period. These findings not only enhance our understanding of the genetic dynamics of the Central Plains but also offer a genetic perspective on the socio-cultural transformations of ancient China during this period. Future studies will incorporate higher-coverage genomic data from larger sample sizes to further explore the complex demographic dynamics and historical changes shaping the genetic landscape of this region.

## Data Availability

The aligned BAM files reported in this study have been deposited in the Genome Sequence Archive in the National Genomics Data (GSA-Human: HRA010900).

## Footnotes

1
https://github.com/stschiff/sequenceTools

2
https://reich.hms.harvard.edu/

3
https://pypi.org/project/hapROH

4
https://www.r-package.org/
