# Supplementary material for: Ancient genomes reveal the genetic structure and population interaction in the Central Plains of China during the Eastern Zhou Period
Source: Front Microbiol. 2025 May 27;16:1602625. doi: 10.3389/fmicb.2025.1602625 (PMC12150079; doi:10.3389/fmicb.2025.1602625)

Supplementary Materials for

**Ancient Genomes Reveal the Genetic Structure and Population Interaction in the Central Plains of China during the Eastern Zhou Period**

**Authors:** Xiyan Wu^1,2,†,^*, Pengxiang Liu^1,2,†^, Fei Yan^3,†^, RongPeng Jin^1^, Canshuo Zhong^1^, Lin Wang^1^, Ke Chen^1^, Fan Yang^1^, Linyi Nie^1^, Yawei Zhou^5,^*, Baoxu Ding^4,^*

^*^Correspondence to: Xiyan Wu(wuxiyan@henu.edu.cn), Yawei Zhou(zhouyawei469@163.com) and Baoxu Ding(dingbaoxu@outlook.com)

**Contents**

1. Figure S1. Ancient DNA damage patterns for the Shangshihe samples.
2. Figure S2. Relationship Estimation from Ancient DNA(READ) assessed genetic relatedness in Shangshihe individuals.
3. Figure S3. Distribution of Runs of Homozygosity (ROH) length (>4 cM) in Shangshihe individuals.
4. Figure S4. PCA result of Shangshihe population with present-day and ancient East Asians.
5. Figure S5. Cross-validation (CV) error estimates of ADMIXTURE for the "HO" dataset.
6. Figure S6. Symmetry F4 statistics in the form of f4(Mbuti.SDG, X; YR-related, Shangshihe) to evaluate the genetic divergence between the Shangshihe population and Yellow River-related populations.
7. Figure S7. Pairwise qpWave analysis between individuals in the Shangshihe cemetery.

**Figure S1. Ancient DNA damage patterns for the Shangshihe samples.** The left represented C to T misincorporations at the 5’ end and the right represented G to A misincorporations at the 3’ end.

**Figure S2.** **Relationship Estimation from Ancient DNA(READ) assessed genetic relatedness in Shangshihe individuals.** Only samples with more than 30,000 SNPs overlapping the 1240K dataset were included in the kinship analysis, which revealed no close genetic relationships among the individuals

**Figure S3. Distribution of Runs of Homozygosity (ROH) length (>4 cM) in Shangshihe sample.** Only samples with more than 100,000 SNPs overlapping the 1240K dataset were included in the ROH analysis. Our findings showed that five individuals (SSH19N, SSH38N, SSH43N, SSH65, and SSH74) exhibited ROH segments exceeding 4 centimorgans (cM). Among them, SSH65 and SSH43N also displayed a limited number of longer ROH segments (>20 cM).


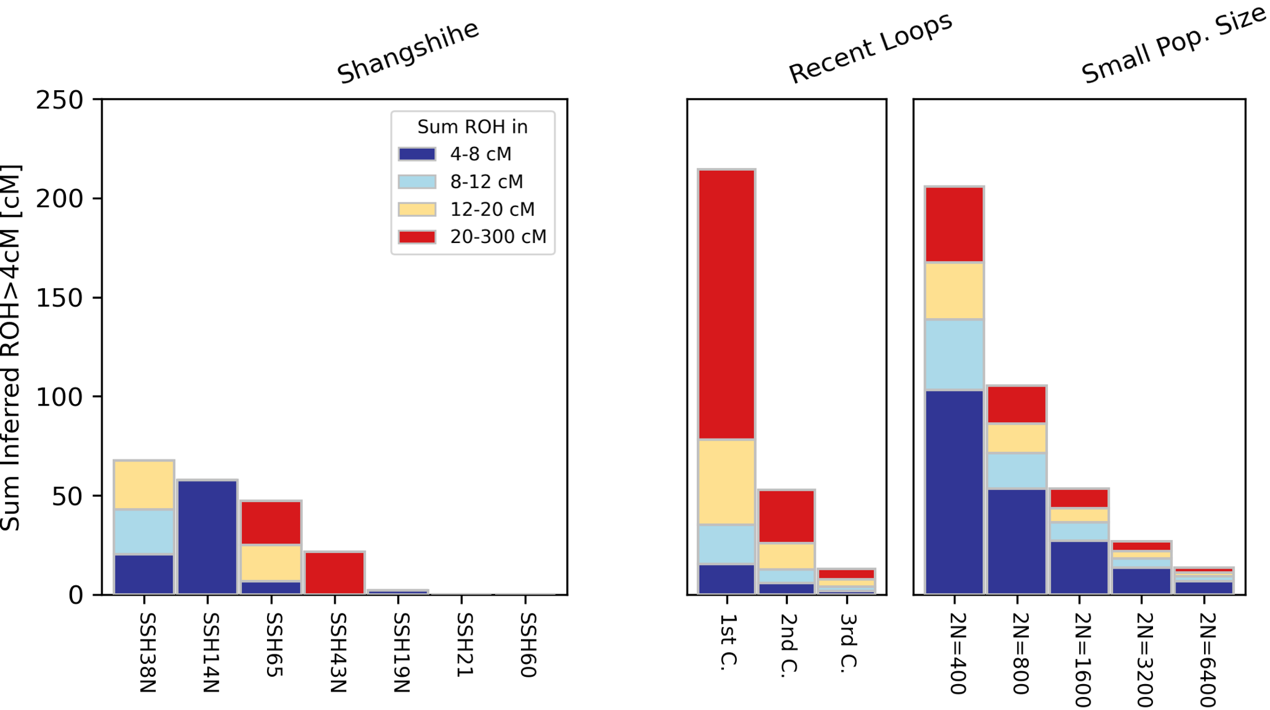


**Figure S4. PCA results of Shangshihe population with present-day and ancient East Asians.** Ancient individuals were projected onto the top PCs.


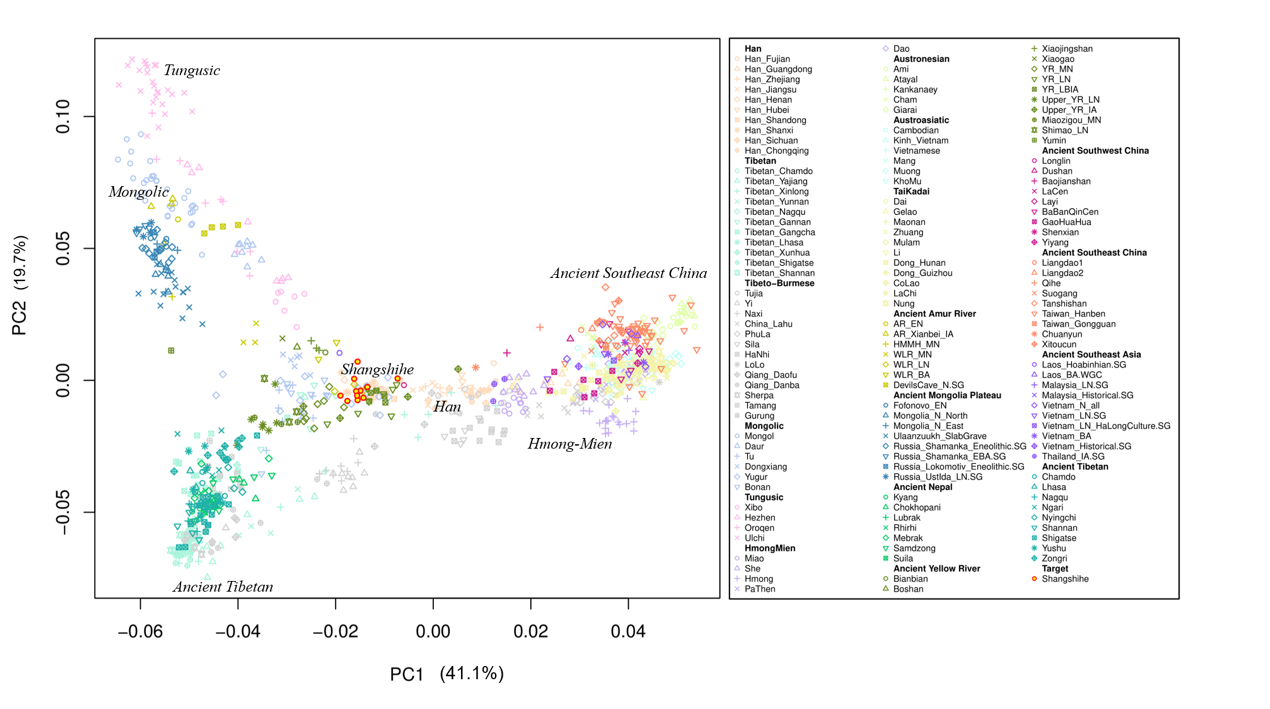


**Figure S5. Cross-validation (CV) error estimates of ADMIXTURE** **for the "HO" dataset.** The K values were ranged from 2 to 20, reaching a minimum at K=5.


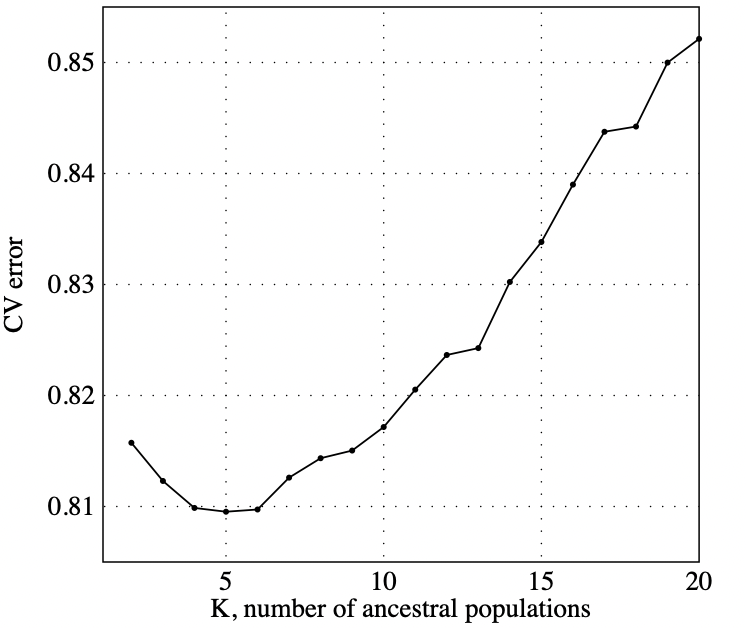


**Figure S6. Symmetry F4 statistics in the form of** **f4(Mbuti.SDG, X; YR-related, Shangshihe) to evaluate the genetic divergence between the Shangshihe population and Yellow River-related populations.** 10 most positive and 10 most negative f4 values are presented. The statistically significant results (|Z | ≥ 2.5) are colored by red.


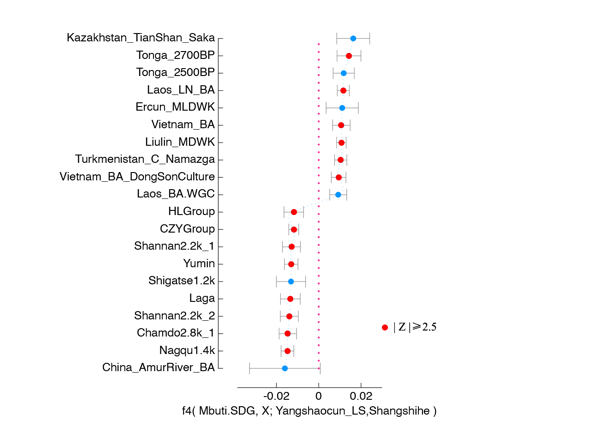

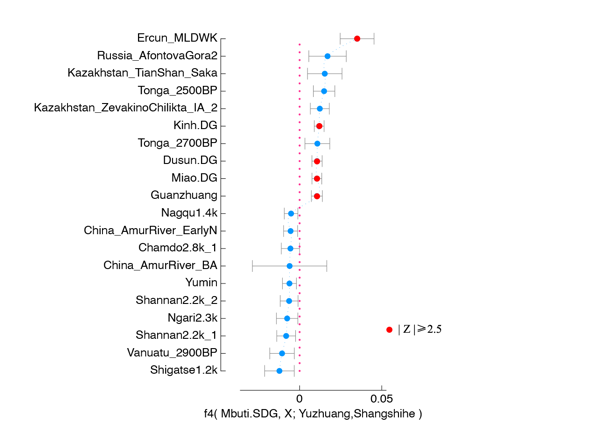


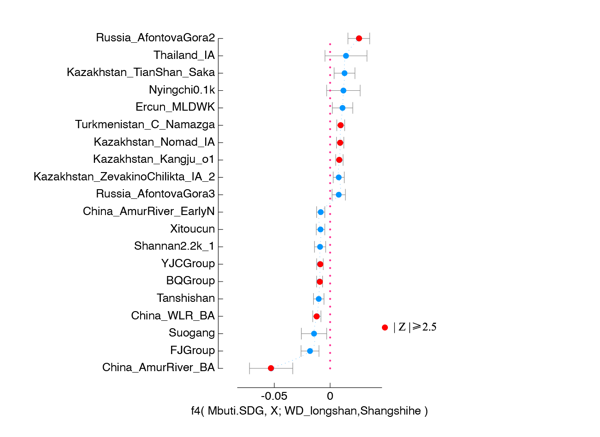

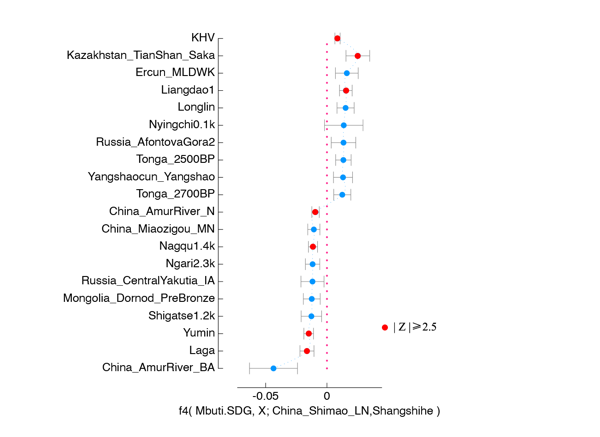


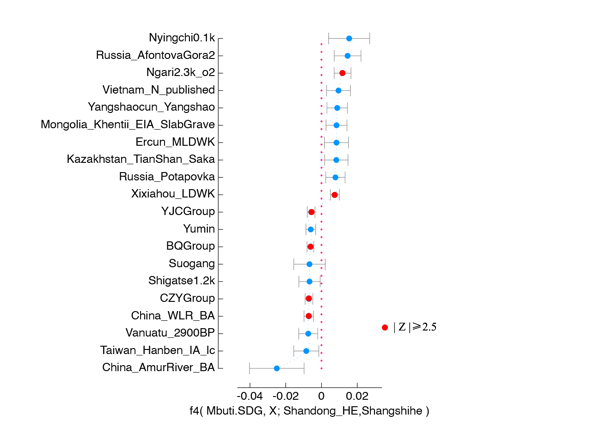

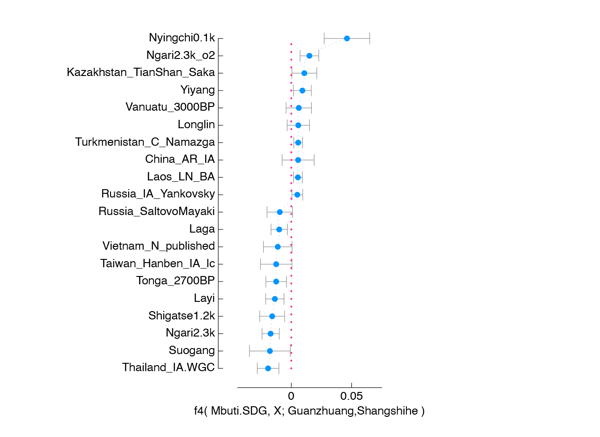


**Figure S7.** **Pairwise qpWave****analysis between individuals in the Shangshihe cemetery.** Samples with less than 40,000 SNPs overlapping the 1240K dataset were excluded from the qpWave analysis. We use a base outgroup, including Mbuti, Onge, Iran _N, Loschbour, Botai, Shamanka, Japan_Jomon, Fujian_EN, Ami, and Shandong_EN. Number in cells represent the p-value (rank = 0) of pairwise qpWave analysis.


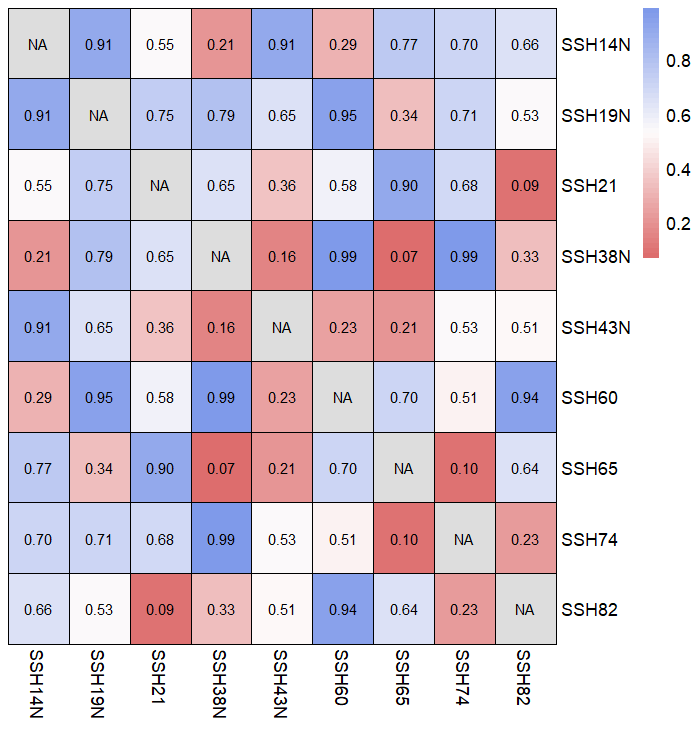

Supplement: Supplementary file 1 [file Data_Sheet_1.docx]
